# Intubing the Larynx for Acute Catarrhal Laryngitis

**Published:** 1886-03

**Authors:** Albert B. Strong

**Affiliations:** Demonstrator of Anatomy, Rush Medical College; 533 West Monroe Street


THE CHICAGO

### MEDICAL JOURNAL AND EXAMINER.

Vol. LII. MARCH, 1886. No. 3.

### Original Communications.

### Intubing the Larynx for Acute Catarrhal Laryngitis. Recovery.

*By* Albert B. Strong, a. m., m. d., *Demonstrator of Anatomy, Rush Medical College.*

[Read before the Chicago Medical Society, February 1, 1886.]

The patient, a delicate child, small for her age of two and one-half years, awoke on Saturday morning, January 17, with a slight cold. She was croupy all day. That night she had decided difficulty in breathing, which the mother relieved with home remedies, causing emesis.

The next morning she played about the house, seemed comparatively well, except that the brazen character of the cough was more pronounced. As evening approached she grew steadily worse. About 6 o'clock Dr. I. N. Danforth was called. He has since made the following note of her condition:

"When I arrived the dyspnœa was very great. With great effort the child could partially expand the lungs, the face was suffused, the eyes were dull, and the child's manner was listless and sleepy, as if partially narcotized. I advised surgical procedure at once."

The narrator saw the child about an hour afterward. Her condition then was as described above.

The diagnosis of acute catarrhal laryngitis was made.

As soon as the parents understood that there was to be no cutting, they readily consented to have the larynx intubed. This was quickly accomplished, tube No. 3 being safely introduced. The labored respiration immediately became natural, and the child passed into a quiet sleep, in which it remained for an hour.

The act of introducing the tube had caused such very free and copious expectoration of frothy, ropy mucus as to apparently, for the time being at least, have effectually cleared the parts of their obstruction. Thinking that the case might do well enough without the tube, and being accessible in case it should not, the tube was removed by drawing upon the thread which had been left hanging from the mouth. The child passed the night fairly well, though occasionally disturbed by the cough. The dyspnœa was present all the time, though not enough to cause much uneasiness to the parents, who had seen croup before.

By four o'clock in the afternoon the dyspnœa had increased to such an extent, in spite of free emesis, that the same tube was re-introduced, and the thread was left protruding from the mouth as before. Large quantities of mucus were again dislodged.

The severe symptoms were instantly relieved and the child slept quietly for two consecutive hours. During the night she was quite restless and uneasy. She did not sleep much, and drank water often, which always excited coughing, followed by free expectoration. Constant care of the nurse was required to prevent the child from removing the tube by pulling upon the thread. This accident did occur in the morning, about twelve hours after the introduction of the tube.

I saw her a short time afterwards. There was yet considerable difficulty both in inspiration and expiration, but still there was no immediate danger, so I concluded not to intube at at present. I left instructions to give an emetic dose of ipecac, should the symptoms require it. I stated that I would call again later in the day. I was summoned in great haste about four hours afterward. I found the little one much prostrated, laboring greatly for breath, with blue lips and clammy extremities. The parents thought we had better let the child alone, as they were sure it would die.

I tried to re-introduce the same tube that had been in position twice before. After several attempts, I was unable to do so, on account of the swelling that had taken place in the glottis. Tube No. 2, the next smaller size, passed in on the first attempt. Again there was free expectoration. In a few moments the thread was removed.

The change in the child was truly remarkable.

The urgent symptoms were relieved at once. From apparent imminent death she passed to a state of assured life. Quiet, refreshing, and undisturbed sleep followed.

During the next sixty-eight hours, which time the child continuously wore the tube, there was nothing of importance to notice, except an entire absence of those symptoms one would naturally expect from the presence of a foreign body in the larynx. During these three days, had any one of you seen the little patient (I am quite sure) you would not have suspected the presence of the tube, except by the absence of the voice.

There was, at times only, moderate coughing with free expectoration. Respiration was natural in every respect, she swallowed solid food readily; fluids not so easily, the latter generally produced slight coughing. Her sleep was comparatively undisturbed. She sat up a good share of the time playing with her toys.

At the end of the sixty-eight hours the tube was removed. From this time respiration was natural. She gained strength rapidly. In the course of a few days her voice had completely returned. Only a moderate amount of fever was present for the first three days. She had but very little medicine. I believe it was of no benefit to her.

Intubing the larynx is an operation, compared to tracheotomy, easily and quickly performed, a few moments of time only being required. It furnishes instant relief without cutting or bloodshed. It is believed to be nearly if not quite free from danger. The consent of parents can be readily obtained. The care of the patient, while wearing the tube, is nothing in comparison with a similar case after tracheotomy. The tube does not have to be removed or interfered with in any way to be cleaned. At least this was so in the case reported. The tube was perfectly free from mucus at the end of sixty-eight hours of continued use. This fortunate condition of affairs, in all probability, depends upon the uniform temperature in the larynx and the canula and inspired air.

The tubes that were first made were easily coughed up. The new ones are so constructed that this will not easily occur, and it did not happen with us. For these reasons I think intubing the larynx will commend itself to general favor, both with the profession and the people.

Removing the tube is much more difficult than introducing it. Could the thread be left attached to the tube and protruding from the mouth its extraction would be an easy matter. There are objections, however, to the presence of the thread. It was observed that during the thirteen hours, while the child wore the tube with the thread attached, she had much difficulty in swallowing, particularly fluids; and she coughed more in proportion than during the three days subsequently, when she wore the tube without the thread.

In other words, the thread seemed to produce the principal part of the irritation. There may be good reasons for this. When the thread is passed through the hole made for it in the tube, at its junction with the anterior part of the collar, and the ends are drawn out of the mouth, it draws across the base of the epiglottis, and so interferes with its accurate mechanical work in closing the glottis. It has seemed to me that if the thread were attached to the posterior part of the collar, on the right side, it would be further removed from the base of the epiglottis and might remedy the evil. I intend to give the idea a trial at the first opportunity I may have. Another objection to wearing the thread, even if it produced no irritation, is that it requires constant watching to prevent the child from pulling the tube out.

On the other hand, one can imagine a piece of false membrane dislodged from the trachea below, and driven against or into the lower end of the tube in such a way as to entirely obstruct respiration. In such a state of affairs the presence of the thread would in all probability be the means of saving that child's life, by quickly removing the tube. It is a matter of some interest then that if the thread is to be left in position it produce the least amount of irritation.

The instruments used and here exhibited are those devised by Dr. O'Dwyer.

The set, complete, consists of five tubes with obturators, an introducing instrument, an extracting one, a gag, and a scale which indicates the tube to be used at a given age. The tubes vary in length from one and one-half to two and one-half inches, suitable for cases from a few months to ten years of age. They are quite heavy, flattened from side to side, plated with gold, having a collar at one end to prevent slipping through the glottis, and a large bulge a little above the middle, that passes below the vocal cords and so prevents its easy expectoration. The tube in the larynx interferes to a surprisingly small degree with the functions of the epiglottis. By means of the thread attached to the tube, the operator removes it easily from the œsophagus or larynx. The obturator, slipped into the threaded tube, is screwed to the introducing instrument. By means of a simple device the obturator is removed when the tube is in position. The extracting instrument has a jointed point that fits loosely into the upper end of the tube, and is made to expand, when the tube can be easily removed.

The gag is the only part of the apparatus that will not work well. It does not expand enough; is easily crowded out by the tongue; though the jaws are lined with lead, it would be very harsh for a baby without teeth. After covering the mouth-pieces with disks of rubber it was found to be very efficient.

Caution is required on the part of the operator that the index finger shall not be wounded by the teeth, since there might be danger of inoculation, in diphtheria.

In the case here reported, to intube the larynx the child was seated on the lap of an assistant, facing the operator; another assistant held the child's head, bending its neck well back. The gag was placed in the left angle of the mouth; the index finger of the left hand held the epiglottis forward and at the same time guided the threaded tube into the larynx. The obturator and gag were at once removed. In a few moments the gag was re-introduced, the index finger placed on the collar of the tube and the thread removed.

To remove the tube, the child was placed in the same position as on introducing it. The point of the extracting instrument was guided by the finger into the orifice of the tube. This was the most difficult part of the whole procedure. No anæsthetic was given.

533 West Monroe Street.

